# Facilitators and barriers in implementation of active TB drug safety monitoring and management (aDSM) in programmatic management of drug resistance TB in Dar es Salaam region

**DOI:** 10.1371/journal.pone.0291225

**Published:** 2023-09-15

**Authors:** Belinda Chriacus Nyaulingo, Francis Apolinary Mhimbira

**Affiliations:** 1 Muhimbili University of Health and Allied Sciences, Dar es Salaam, Tanzania; 2 Kibong’oto Infectious Diseases Hospital, Kilimanjaro, Tanzania; The University of Georgia, UNITED STATES

## Abstract

**Background:**

World Health Organization (WHO) recommends that active TB Dug Safety Monitoring and Management (aDSM) be adopted in countries’ programmatic management of DR-TB services. In Tanzania, the National TB Leprosy Programme (NTLP), under the ministry of health, adopted the aDSM component in 2018. The study evaluated the facilitators and barriers of aDSM implementation in Dar es Salaam.

**Materials and methods:**

This was a process evaluation study that adapted the descriptive cross-sectional approach, conducted in Dar es Salaam region. A total of 19 respondents, including clinicians, DOT (Direct Observed Therapy) nurses and key NTLP personnel, were interviewed using interview guides. Qualitative content analysis based on Graneheim & Lundman was used to guide the analysis.

**Results:**

For aDSM to be implemented in a health facility, tools like forms for recoding and reporting, access to a functional laboratory for carrying out the required monitoring tests are a necessity. Moreover, the NTLP monitors the implementation through received aDSM reports and DR-TB supportive supervisions. However, it was found that in many health facilities, aDSM was partially being implemented due to various barriers: inadequate trained staff for aDSM implementation, administrative burden in reporting and delaying in AE management.

**Conclusion:**

aDSM is inadequately being implemented due to the many setbacks faced by HCWs. aDSM-specific supportive supervisions and trainings to HCWs; incorporating the current manual aDSM reporting flow into the already existing electronic (Tanzania Medicine and Medical Drugs Authority) TMDA database seems useful.

## Introduction

Multidrug-resistant tuberculosis (MDR-TB) is a public health crisis and a global health security risk carrying grave consequences for those affected. Globally in 2021, there were an estimated 450,000 (95% uncertainity level: 399 000–501 000) incident cases of MDR/RR-TB and an estimated 182,000 deaths from DR-TB [1]. In Tanzania in 2018, there were 449 notified incident DR-TB cases among which 409 (91%) were enrolled for programmatic management of DR-TB treatment (DR-TB) services [2]. Patients with rifampicin-resistant or Multidrug-resistant tuberculosis (MDR-TB) are treated with a combination of second-line drugs making up a regimen that can either be standardized shorter or individualized regimens. At least 70% of DRTB patients experience one or more Adverse Events (AEs during treatment may lead to default from treatment and result in poor treatment outcomes) [3,4]; thus, some patients default from treatment and results in poor treatment outcomes. In 2015, the WHO introduced active TB drug-safety monitoring and management (aDSM); a framework to facilitate the active pharmacovigilance (PV) for new and repurposed drugs to be conducted in a less labour-intense form. The active Drug Safety Monitoring and Management (aDSM) component in the programmatic management defines active and systematic clinical and laboratory assessment of patients while on treatment. aDSM applies to patients on treatment with new anti-TB drugs, novel MDR-TB regimens or extensively drug-resistant TB (XDR-TB) regimens, in order to help detect, record and report, manage and monitor the anticipated adverse events [5]. By the end of 2018, the WHO aDSM guidelines were adapted for the Tanzania context in national guidelines and subsequently adopted by 111 health facilities (the total number of health facilities that had enrolled patients for MDRTB treatment in the whole country) across the country [6].

DR-TB practitioners are required to immediately report a Serious Adverse Event (SAE) through the regional aDSM focal person then national focal person, so that it reaches the Uppsala centre under WHO within 24 hours after its occurrence. They’re also required to report all AEs of special interest on a monthly basis, following the same manner as with SAE reporting. This reporting is done through special aDSM forms. More importantly, they’re required to manage and closely monitor the occurred AEs/SAEs until their resolution. However, the spontaneous reporting rarely occurs in practice [7].

In accordance with the WHO aDSM recommendations, countries implementing the aDSM framework are required to ensure availability of national guidance document on detection, clinical management and reporting of AEs [8], availability of recording and reporting aDSM tools and AE report flow from facility level to national and to global levels. In countries like Vietnam, Kyrgyzstan and Ethiopia schedules for national level supportive supervision visits and (distance) mentoring have also been set in addition [9]. Several countries have reported setbacks during aDSM implementation: Unsustainable availability of appropriate tests for diagnosing the Adverse Events (AEs); Insufficient knowledge of HCWs on how to detect, manage & monitor the adverse events; The already trained HCWs not recording and reporting the AEs as recommended. The speculated explanation for this AE under-reporting includes lack of awareness and administrative burden (need to report to the country pharmacovigilance centre and to the aDSM system with different forms and multiple steps); Unsustainable availability of medications for AEs management and patients [8–10]. In Sub-Saharan African countries, the identified challenges were as follows: Low capacity of DR-TB clinicians in treatment sites to measure and interpret ECGs and audiometry; Poor internet access at most treatment initiation sites hinders reporting of SAE within 24-hour period; National aDSM technical working group not regularly involved in aDSM activity planning; Limited staff and lack of capacity for causality assessment at National Pharmacovigilance Centres (NPVC) [10,11].

In Tanzania, it is uncertain to what extent aDSM is being implemented, in accordance with the aDSM policies and guidelines, in health facilities caring for DR-TB patients. Therefore, the study aimed at evaluating the facilitators and barriers of aDSM implementation so as to fill the gap of this scarce information.

## Materials & methods

### Context of the study

Tanzania has 31regions with Dar es Salaam included. It is the largest business city and the smallest region geographically and yet the most populated region in the country. Due to this congestion Dar es Salaam has the highest number of DR-TB patients across the country in accordance with the Electronic TB & Leprosy register (ETL 2020 reports).

The provision of programmatic management of DR-TB services is available at all levels of health facilities in the country: from primary level (consisting of health centres, dispensaries and district hospitals) to tertiary level (consisting of zonal hospitals, consultant hospitals, specialized hospitals and the national hospital).

The study was conducted in all five districts of Dar es Salaam region: Ilala, Temeke, Ubungo, Kinondoni and Kigamboni; and at the national level, the National Tuberculosis & Leprosy Programme (NTLP) under the ministry of health.

### Study population

The study involved all health care workers (DOT nurses and clinicians), who are the implementers of aDSM, in health facilities with DR-TB clinics that enrolled DR-TB patients for management in the year 2020; and the national aDSM focal person and national DR-TB coordinator from the NTLP who are the enforcers of the aDSM set policies and guidelines [**Table 1**].

**Table 1:** Study participants (in depth interviews).

Study participants	Number (in public health facilities)	Number (in private health facilities)	Total number
DOT nurses	7	1	8
Clinicians	9	0	9
National aDSM focal person	1	Not applicable	1
National DR-TB coordinator	1	Not applicable	1

### Sampling strategy

At the national level, the NTLP, a purposeful sampling strategy was used to obtain key informants. The directorate of human resource was consulted so as to identify the respondents that were involved with the enforcement of aDSM implementation; this involved the national DR- TB coordinator and the national aDSM focal person.

A purposeful sampling procedure was used to obtain all health facilities that had enrolled DR-TB patients in the 2020. The enrolment started by identifying these facilities through the Electronic Tuberculosis & Leprosy Register (ETL register), run by the NTLP; a total of seventeen facilities (hospitals, dispensaries and health centres) were obtained. At these health facilities, participants (DOT nurses and clinicians) were obtained by convenience sampling strategy. Those who were present during data collection period and those who agreed to participate in the study were included.

### Data collection

Data for this study was collected between May and June of 2021. Before data collection, the researcher contacted the selected informants via phone call to set up the appointment for the interview. For the DOT nurses and clinicians, the medical officers in charge in the respective facilities were contacted via phone call to inform and organize the respondents. During data collection, the in depth interviews were carried out by the researchers. Both audio files and transcripts were stored in a computer where only the researcher had access. Each interview took around 25–35 minutes.

### In-depth interviews

Two different semi-structured interview guides were used for HCWs and the other for the informants from NTLP to carry out 17 in depth interviews [**Table 1**]. The questions in the interview guides were formulated based on experiences as documented in already existing literature.

### Data analysis

Interviews were transcribed verbatim. The Kiswahili transcripts were then translated into English before analysis. Qualitative content analysis based on Graneheim & Lundman was used to guide the analysis [12]. The text data was repeatedly read until a sense of the whole was achieved. From the condensed meaning units, exact words were highlighted and labeled as the initial codes. After which labels for codes, derived directly from the text data, reflecting more than one key thoughts/concepts are formed.

Then similar codes were grouped together through abstraction to form sub-categories. The sub-categories were grouped into categories based on how the codes are related to reflect the manifest content of the text, which was backed up with suitable quotes from the transcripts. Finally, the definitions of the categories were developed to ensure that the latent meaning was also brought into focus. The whole process was iterative to ensure that both the manifest and latent meaning of the data is not lost.

### Ethical considerations

The ethical clearance was obtained from the Institutional Review Board (IRB), named Muhimbili University of Health and Allied Sciences Ethical Review Committee of Research and publication. The permission to conduct the study in the hospitals was approved by the regional and district medical officers, medical officers in charge for public and private hospitals; the NTLP Program manager for conducting the study at the NTLP offices.

In addition, before commencing of data collection, an informed written consent was sought from the participants and only those who gave their informed consent were allowed to participate in the study.

## Results

From the interviews, two categories of facilitators and three categories of barriers in aDSM implementation were revealed. The facilitators were: adequacy of non-human resources and provision of technical support. The barrier categories were: inadequate trained staff for aDSM implementation, administrative burden in reporting and delaying in AE management [**Table 2**].

**Table 2:** Facilitators and barriers of aDSM implementation in Dar es Salaam region.

THEMES	CATEGORIES	SUB-CATEGORIES
Facilitators of aDSM implementation	Adequacy of non-human resources	• Presence of forms for recording and reporting the AEs • Availability of investigation tools for toxicity monitoring tests • Presence of AE management in DR-TB guideline
	Provision of technical support	• Accessibility of a sample referral system • Capacity building to HCWs • Presence of online platform for AE management consultation • Financial support for regional Pharmacovigilance activities
Barriers of aDSM implementation	Inadequate trained staff for aDSM implementation	• Few HCWs in DR-TB clinics • Shifting of trained staff to other departments • DOT nurse doing patient consultation • Unsystematic handing over of duties among HCWs
	Administrative burden in reporting	aDSM reporting is still manual • Little commitment among HCWs
	Delaying in AE management	• Delaying in AE recording • Sample referral system inaccessible in private health facilities • Delaying in AE management due to late arrival of results

### Adequacy of non-human resources

HCWs reported that having access to a functioning laboratory and other equipment like ECG machine in order to carry out the necessary toxicity monitoring tests was necessary; without such they are left unable to detect, monitor and manage the suspected adverse events to patients. They stressed on the importance of such laboratories being within their facility so that they can implement aDSM on a timely basis.

“*…The necessary tools required are the availability of laboratory investigations so that the patient is monitored through tests especially ECG*, *FBP*, *RFT*, *LFT…”*

(FACILITY D1)

Most health care workers reported that in order for them to implement aDSM at their facilities, forms used to record and report the occurring adverse events to patients are usually present.

“*…There’s the special aDSM SAE/AE form that we use after we suspect an adverse event in a patient and there’s the nurse’s Daily DOT form*.*”*

(FACILITY D2)

The participants revealed that there are guidelines in health facilities to help HCWs to abide to while implementing aDSM. Such guidelines and SOP help the health care workers to properly carry out aDSM and it is through these that the NTLP checks that the standard for aDSM implementation is maintained and in one accord throughout the country.

“*…We have the DR-TB guideline that has a section on aDSM implementation*.

In addition, we have just finished formulating the National TB & Leprosy Strategic Plan and aDSM implementation has been incorporated in one of the agendas…”

(NTLP A2)

### Provision of technical support

It was discovered that despite the fact that most health facilities did not have a functioning laboratory with all the necessary toxicity monitoring tests; there was a sample referral system in place where on a monthly basis and when need be, samples for investigations are sent to Muhimbili instead. The referral system is supported by the NTLP so as to ensure that at the health facility level aDSM implementation is feasible.

As one respondent reported:

“*The laboratory tests that are done at Muhimbili means the national program facilitate this so that we get reliable results*.*”*

(FACILITY F2)

From the study, it was found out that HCWs are able to seek consultation when encountering difficult adverse events during aDSM implementation. They use the available online platform through zoom meeting, the TB ECHO that is conducted on a weekly basis. Some health care workers also reported that they easily have access to national TB consultants through mobile calls. Both of these methods enable them to implement aDSM even when encountering obstacles.

“*…If we have a patient’s case that we need help in managing*, *we can present it in zoom meetings during DR-TB ECHO sessions*…*”*

(FACILITY C1)

It was revealed that trainings and supportive supervisions are conducted so as to enhance aDSM performance improvement among HCWs by re-orienting them with the forgotten aDSM skills and procedures, identifying implementation challenges and providing them with new knowledge/skills.

“*…They also visit us during supportive supervisions where they check and follow up on us*

*But they also do mentorships*, *mostly during supervisions*…”

(FACILITY F2)

### Inadequate trained staff for aDSM implementation

Most informants stated that there is few HCWs in DR-TB clinics in relation to the number of DR-TB patients making it difficult for them to timely record, report and monitor the AEs. In absence of the clinician, the nurse is thus forced to attend the patients’ AEs. Some reported that, only a few of HCWs in DR-TB clinics have been trained in the aDSM component.

“*…It happens that there are times you have other duties to complete too so after seeing and attending the patient you may decide that you’ll jot down the patients’ notes later but you eventually end up not doing so because of being occupied with other duties… Again*, *sometimes patients come in and I’m not around and they’re thus attended by the DOT nurse*… *So*, *upon coming back*, *I sometimes find out that maybe that patient had an adverse event since the previous week/weeks when they were seen by other clinicians or the DOT nurse but it wasn’t reported…”*

(FACILITY C1)

Other respondents reported that there’s high staff turn over those results in further depletion of the already few trained HCWs in aDSM. Some stated that, once they shift to other departments, their job descriptions are left unattended and this further hump the implementation of aDSM.

*The main challenge is that our aDSM regional focal person was given other duties while he was the one who was trained specifically for aDSM*; *as of now we don’t know where to send our SAE/AE reports since we don’t have the focal person to send them to*.

(FACILITY I1)

### Administrative burden in reporting

Informants reported that HCWs have little commitment in aDSM implementation especially when it comes to recording and reporting the occurred adverse events of patients. Some declared its because the recording and reporting is manual and this requires them to fill in forms and then scan them with their own mobile devices then send them via email to the higher level, this is time consuming and so they choose not to. Others stated that the same AE has to be reported twice-using different forms for TMDA and NTLP.

“*… Little commitment of HCWs in recording and reporting of the AEs; incomplete reports from the health facilities to the extent most of them don’t make it to the TMDA; high staff turnover is a threat to the capacities we have already built resulting in having inexperienced HCWs not fully implementing aDSM; aDSM reporting is still manual since HCWs have to scan the report forms then send the scans via email*.*”*

(NTLP B3)

Some declared it’s because it’s not part of their primary job description and therefore they tend to do it after they’re done with their daily duties, if at all they have spare time.

*You find that most times I am alone at the clinic*, *so when I am with the patient and want to document their details on the monthly follow up form*, *I maybe summoned to go to the wards and attend to other patients*… *At this point I am unable to proceed filling in the form and I dismiss the patient and ask to come in their next visit*.

(FACILITY C2)

### Delaying in AE management

Respondents stated that patients in private health facilities have to incur costs for the toxicity monitoring tests, unlike those in public health facilities where the sample referral system is in place.

*The main challenge is that because our [private health facility] patients have to pay for the baseline and follow up investigations*, *… sometimes we delay in starting their management because most of them are not financially well and we have to wait until they are able to pay for the investigations*.

(FACILITY H2)

Other respondents reported that they mostly receive the toxicity monitoring results facilitated by the sample referral system at a delay and this causes them to further delay in managing the patients’ AEs

*We receive the laboratory results from Muhimbili*, *at a delay… and this is a challenge because the results don’t come as early as we expect them to*. *When you have a new patient*, *you need the baseline results in order for you to know what regimen will best for the patients*, *so if results come in late*, *it means we’re delaying management to the patient*.

(FACILITY D1)

## Discussion

Most health facilities were found to have the necessary forms for recording and reporting AEs (Adverse Events) and DR-TB guidelines to help facilitate in the management of the AEs. In order for the HCWs to timely detect and confirm the AEs, they declared that they must have access to monitoring equipment like the ECG machine and a functional laboratory at the health facility that is able to test all the necessary toxicity monitoring tests. From the findings however, almost all health facilities did not have a functional laboratory in site, but there was a sample referral system instead that was coordinated by the program (NTLP). These results are similar to a study done in Tanzania where implementation of aDSM was found to be difficult since most health facilities either lacked the necessary toxicity monitoring equipment like ECG, or had been faulty or HCWs were unable to operate it [13]. Another similar study conducted in India, on adverse events among patients being programmatically treated, found that guidelines, SOPs and aDSM forms were available at the treatment centre; however some toxicity monitoring tests were not done due to absence of machines for the respective tests [14]. With facilitation of toxicity monitoring tests being far from adequate, the program should increase number of functional laboratories at least one laboratory in each district; rather than having just the Muhimbili national laboratory to serve all health facilities in Dar es Salaam region. Having a functional laboratory in each district under the sample referral system will help ensure the timely provision of monitoring test results that will hence cause the HCWs to manage the AEs without delay.

Less than half the numbers of clinicians and DOT-nurses were found to have been trained on aDSM component. This means very few health care workers had awareness on adverse events management, recording and reporting; a finding coherent with a study conducted in Namibia [15].Similar findings were seen in a study done India where it was reported that lack of training in AE management resulted in health care workers not knowing what, when or why they should report the adverse events occurring to the patients they attended [14]. The low number of trained staff in these findings could be attributed by the limited funds allocated for pharmacovigilance activities and high staff turnover among departments in the health facilities.

Most HCWs have little commitment and would rather not report the AEs since it is considered an administrative burden i.e., they have to report the same AE to NTLP and Tanzania Medicine and Medical Devices Authority (TMDA) using different forms and each involving several steps. This finding is similar to a study done in Ethiopia that showed that the aDSM report flow has complex reporting requirements which then results in duplication of work and eventually leading to late reporting and incomplete/ low quality reports [9]. These findings encountered during reporting could be attributed by the fact that aDSM reporting, at the health facility level, is manual rather than being electronically based. Not only that, but also be attributed by the fact that the aDSM reporting flow does not link information between NTLP and the TMDA who are the enforcers of pharmacovigilance activities in the country.

Patients in private health facilities were found to be paying for all their toxicity monitoring tests as far as aDSM and DR-TB management is concerned; contrary to those receiving DR-TB management from public facilities. This finding could have been attributed by the fact that very few newly diagnosed DR-TB patients go to private health facilities for management, thus the program ended up introducing the sample referral system to where most patients get their services, in public health facilities. However, the program should extend the referral system even to the private health facilities so as to ensure that the financial burden is lifted off the patients’ shoulders. This will result in these patients being correctly monitored as far as aDSM is concerned.

### Methodological consideration

The Lincoln and Guba’s Four Criteria were used to enforce the trustworthiness of the findings of the study: credibility, transferability, confirmability and dependability [12]. The credibility of this study was obtained through the triangulation of informants who had rich information on the study questions. Data were collected from DOT nurses and clinicians from both public and private health facilities. To confirm that the findings represented informants’ thoughts rather than the researchers’ understanding of the questions under study, categories were inductively generated using content analysis and presented with the support of sub-categories and quotes. The transferability of the study was obtained through the description of the study setting, context, data collection process and analysis.

Since it was conducted in Dar es Salaam, an urban setting, the study may not have captured practices in health facilities found in rural areas. We thus recommend that further studies should be done in rural areas to elicit their views. Secondly, social desirability may have limited the findings of this study, as medical doctors led data collection and thus the HCWs may have felt that they should provide desired answers instead of truth-value. However, triangulation of researchers offset this limitation.

## Conclusion

The findings in the study revealed that, despite Dar es Salaam being the region in the country with the highest number of DR-TB patients, it had few trained health care workers and this resulted in aDSM implementation being minimally practiced. Little commitment among HCWs was vividly reflected in the under-reporting of the AEs. In addition, having to report the same AE twice using different forms (TMDA and NTLP aDSM forms) appeared to be an administrative burden to health care workers; thus, some would report to either TMDA or NTLP or not to any at all.

## Supporting information

S1 File (DOCX)
