# Supplementary material for: Facilitators and barriers in implementation of active TB drug safety monitoring and management (aDSM) in programmatic management of drug resistance TB in Dar es Salaam region
Source: PLoS One. 2023 Sep 15;18(9):e0291225. doi: 10.1371/journal.pone.0291225 (PMC10503757; doi:10.1371/journal.pone.0291225)
Supplement: S1 File — (DOCX) [file pone.0291225.s001.docx]

**SUPPORTING INFORMATION FILE**

**DATA REDUCTION MATRIX**

| **Domain** | **Initial codes** | **Codes** | **Sub-categories** | **Categories** | **Condensed meaning units (selected quotes)** |
| --- | --- | --- | --- | --- | --- |
| Facilitators of aDSM implementation | -There’s the special aDSM SAE/AE form  - Used after we suspect an adverse event in a patient  - There’s the nurse’s Daily DOT form  - Monthly follow up form  - aDSM adverse events management SOP  -Availability of laboratory investigations  -ECG machine  - There’s the yellow and green forms  - Check to what extent are the adverse events  -There’s the aDSM SAE/AE forms  -DR-TB guideline  - National TB & Leprosy Strategic Plan | **-** aDSM SAE/AE form  **-** Nurse’s Daily DOT form  - Monthly follow up form  - Yellow and green forms  -Laboratory investigations  **-** ECG machine  - aDSM adverse events management SOP  **-**DR-TB guideline  -National TB & Leprosy Strategic plan | Presence of forms for recording and reporting the AEs  Availability of investigation tools for toxicity monitoring tests  Presence of AE management in DR-TB guideline. | Adequacy of non-human resources | “The necessary tools required are the availability of laboratory investigations so that the patient is monitored through tests especially ECG, FBP, RFT, LFT…” (*FACILITY D1)*  “…There’s the special aDSM SAE/AE form that we use after we suspect an adverse event in a patient and there’s the nurse’s Daily DOT form.”  (*FACILITY D2)*  …aDSM adverse events management SOP that guides us on how to manage the AEs that have occurred to a patient and the ECG machine. (*FACILITY F2)*  There’s the monthly follow up form; there’s the yellow and green form that check to what extent are the adverse events and then there’s the aDSM SAE/AE forms. (*FACILITY I1)*  We have the DR-TB guideline which a has a section on aDSM implementation.  In addition, we have just finished formulated the National TB & Leprosy Strategic Plan and aDSM implementation has been incorporated in one of the agendas. (NTLP A2) |
|  | - Laboratory tests are done…national program facilitates this  -…visit us during supportive supervisions…they also do Mentorships  - during these trainings there’s usually the aDSM component that is taught.  - Consult the higher level for proper management  -Prevent the adverse event from worsening  -A patient’s case need help in managing  -Present it in zoom meetings during DR-TB ECHO sessions  -Training of the regional aDSM focal person  -Fund allocation for regional Pharmacovigilance (PV) interventions | -Toxicity monitoring tests are done  -Supportive Supervisions  -Mentorships  -Trainings  -Patient case consultation in DR-TB ECHO sessions  -Funds for PV interventions  - Training in aDSM implementation among regional aDSM focal person | -Accessibility of a sample referral system  -Capacity building of HCWs  -Presence of online platform for AE management consultation  Financial support for regional PV activities | Provision of technical support | The laboratory tests that are done at Muhimbili means the national program facilitate this so that we get reliable results... They also visit us during supportive supervisions where they check and follow up on us.  But they also do mentorships, mostly during supervisions. (*FACILITY F2)*  Yes there’s support. Example we get trainings and during these trainings there’s usually the aDSM component that is taught. (*FACILITY D2)*  If we have a patient’s case that we need help in managing, we can present it in zoom meetings during DR-TB ECHO sessions. (*FACILITY C1)*  There’s fund allocation for regional Pharmacovigilance (PV) interventions, mentorships and supportive supervisions to health facilities, to be done by the regional aDSM focal person; and lastly as a program we have supportive supervisions. *(NTLP B3)* |
| Barriers of aDSM implementation | -Occupied with other duties  **-** Sometimes patients come in and I’m not around  -Attended by the DOT nurse  **-** Unless they have a complaint that requires a doctor’s review then the DOT nurse can take that patient to other clinicians available  **-** Most times I am alone at the clinic  **-** At this point I am unable to proceed filling in the form and I dismiss the patient and ask them to come in their next visit  **-**Results come in late  **-**Delaying management to the patient  -Sustainability of the investigations  -Some investigations… are not covered by the program  -Patient have to incur costs  -Most patients have low economic statutes  **-…** Patients have to pay for the baseline and follow up investigations  - Sometimes we delay in starting their management because most of them are not financially well and we have to wait until they are able to pay for the investigations  -The main challenge is that our aDSM regional focal person was given other duties while he was the one who was trained specifically for aDSM  - as of now we don’t know where to send our SAE/AE reports since we don’t have the focal person to send them to.  - We’ve only been able to train a few of them  -Little commitment of HCWs in recording and reporting  -Incomplete reports from the health facilities to the extent most of them don’t make it to the TMDA  - High staff turnover is a threat to the capacities we have already built  - Having inexperienced HCWs not fully implementing aDSM  - aDSM reporting is still manual | -Heavy work load  -Few HCWs  -Delaying in AE recording  -Few HCWs  -Unequal work division  -Prolonged turn around time in the sample referral system  -Delaying AE management to patients  -Unsustain  ability of toxicity monitoring tests  -Sample referral system only covers some investigations  Delaying due to late arrival of results and unsustainable investigations  - Delay in starting DR-TB management  - Unable to pay for investigations  - Shifting of already trained staff  - No proper handing over of duties  -Few trained HCWs  - Trained HCWs shifting to other departments  -Untrained  HCWs cannot fully implement aDSM  -Manual aDSM reporting | -Delaying in AE recording  -Few HCWs in DR-TB clinics  - Shifting of trained staff to other departments  -Delaying in AE management due to late arrival of results  -Sample referral system inaccessible in private health facilities  -Patients unable to pay for toxicity monitoring tests  -Unsystematic handing over of duties  -aDSM reporting is still manual  -DOT nurse doing patient consultation  -Little commitment | -Inadequate trained staff for aDSM implementation  -Delaying in AE recording  -Delaying in AE management  -Patients paying for toxicity monitoring tests  -Admini-  strative burden in reporting | “…It happens that there times you have other duties to complete too so after seeing and attending the patient you may decide that you’ll jot down the patients’ notes later but you eventually end up not doing so because of being occupied with other duties… Again sometimes patients come in and I’m not around and they’re thus attended by the DOT nurse unless they have a complaint that requires a doctor’s review then the DOT nurse can take that patient to other clinicians available. So upon coming back, I sometimes find out that maybe that patient had an adverse event since the previous week/weeks when they were seen by other clinicians or the DOT nurse but it wasn’t reported…”  (FACILITY C1)  “You find that most times I am alone at the clinic, so when I am with the patient and want to document their details on the monthly follow up form, I maybe summoned to go to the wards and attend to other patients or maybe there’s an emergency. At this point I am unable to proceed filling in the form and I dismiss the patient and ask them to come in their next visit…”  (FACILITY C2)  We receive the laboratory results from Muhimbili, … and this is a challenge because the results don’t come as early as we expect them to. When you have a new patient, you need the baseline results in order for you to know what regimen will best for the patients, so if results come in late it means we’re delaying management to the patient.  *(FACILITY D1)*  There are some investigations like ultrasound that are not covered by the program and thus the patient has to incur costs when such an investigation is needed. *(FACILITY E1)*  The main challenge is that because our [private health facility] patients have to pay for the baseline and follow up investigations, …sometimes we delay in starting their management because most of them are not financially well and we have to wait until they are able to pay for the investigations *(FACILITY H2)*  The main challenge is that our aDSM regional focal person was given other duties while he was the one who was trained specifically for aDSM; as of now we don’t know where to send our SAE/AE reports since we don’t have the focal person to send them to.  (ACILITY I1)  There’s knowledge gap among health care workers (HCWs) since we’ve only been able to train a few of them; little commitment of HCWs in recording and reporting of the AEs; incomplete reports from the health facilities to the extent most of them don’t make it to the TMDA; high staff turnover is a threat to the capacities we have already built resulting in having inexperienced HCWs not fully implementing aDSM; aDSM reporting is still manual since HCWs have to scan the report forms then send the scans via email.  *(NTLP B3)* |
